# Douching: A Risk to Women's Healthcare?

**DOI:** 10.1080/10647440300025511

**Published:** 2003

**Authors:** Mark Martens, Gilles R. G. Monif

**Affiliations:** Department of Obstetrics and Gynecology University of Oklahoma –Tulsa 1145 South Utica Suite 600 Tulsa OK 74104 USA


*Infect Dis Obstet Gynecol 2003;11:135–137*

### Douching: a risk to women's healthcare?

**Mark Martens and Gilles R. G. Monif**

*Department of Obstetrics and Gynecology, University of Oklahoma – Tulsa, Tulsa, OK*

A number of cross-sectional, static-group studies have suggested that douching can be linked to bacterial vaginosis (BV) in pregnant women, non-pregnant women attending sexually transmitted disease (STD) clinics, and non-pregnant female sex workers in Nairobi[1–4]. Gresenguet and co-workers[5] advanced data which they interpreted as linking human immunodeficiency virus acquisition to douching. Other studies have cited a linkage between douching and pelvic inflammatory disease, preterm labor, preterm delivery and cervical cancer[6–9].

The vast amount of federal funding to support, in particular, the Seattle groups[10–16] has attested to the perceived importance of this issue for women's healthcare. Douching is a common practice for which there is little scientific data to support or repudiate the stated allegations. Nevertheless, serious discussions have been held by the Food and Drug Administration as to the banning of commercial douching preparations. If this is done, it must be based on valid scientific experimentation.

Concern has been raised as to an ongoing failure to control critical variables and to the experimental designs chosen. The studies suggesting a causal relationship have been primarily static-group comparisons and/or pseudo- or quasi-experimental designs[17,18]. While these designs can effectively pose the hypothesis, they do not constitute a validating scientific vehicle.

To look critically at the control of potentially invalidating variables for douching, one needs to begin with those which impact on BV study populations. Identical claims of causality (pelvic inflammatory disease, ectopic pregnancy, secondary infertility, preterm births) have been advanced for BV as have been claimed for douching. Rosenberg and co-workers[9] had identified three characteristics that stood out among women who douched: lower socioeconomic status, greater risk of sexually transmitted diseases and symptoms suggestive of vaginal infections. Multiple studies of women with BV have revealed a high incidence of co-infections with other STD pathogens and, in particular, *Chlamydia trachomatis*[10,11,19–23]. No mechanism has been demonstrated how douching causes pelvic inflammatory disease, ectopic pregnancies, secondary infertility; however, such data does exist for *Chlamydia trachomatis*, in the absence of douching.

Recently, Ness and co-workers reported on a multi-institutional National Institutes of Health (NIH)-funded project which studied 1200 women at high risk for STDs in order to document a possible association between douching and BV[24]. Women were eligible for the study based on a previous risk stratification for chlamydial cervicitis, which introduces a strong bias into the results. The study conclusions represent a conceptual change in that, rather than douching causing the long-term sequelae to women, douching causes BV which in turn causes the stated outcomes. The investigators proposed that douching predisposes to BV by disrupting the normal vaginal flora.

They identified, as other studies have documented, that women of black race, low education, low income and a history of STDs were more likely to have BV than normal flora (76.5% versus 19.1%). Women who were never pregnant, smoked and had sexual intercourse with menses

Correspondence to: Mark Martens, MD, Professor of Obstetrics and Gynecology, University of Oklahoma College of Medicine – Tulsa, 1145 South Utica, Suite 600, Tulsa, OK 74104, USA. Email: gmonif@aol.com

© 2003 The Parthenon Publishing Group 

were also more likely to have BV. Douching once a month or more elevated the risk of having BV or what they termed 'intermediate vaginal flora' (IVF) by a factor of 1.4. Those women who found it necessary to douche within the past 7 days were at even higher risk of having BV or IVF. Women with BV or IVF had a higher prevalence of gonococcal or chlamydial cervicitis.

The theory that douching disrupts the normal vaginal flora and selects for BV has little, if any, microbiologic foundation as it involves the effects of douching. To date, there are only two studies with douching which possess sequential quantitative and qualitative microbiologic observations. Onderdonk and co-workers[25] evaluated the quantitative and qualitative effects of two douching preparations on the vaginal microbial flora. The 0.04% acetic acid douche caused transient reduction in the total bacterial count which was attributed to a wash-out effect. The 0.3% povidone–iodine preparation confirmed the earlier work of Monif and co-workers[26]; it caused a statistically significant reduction in the total bacterial counts when compared to physiologic saline in the same subjects. Monif and co-workers have demonstrated that povidone–iodine gel caused a dramatic, but transient elimination of bacterial isolates from the female genital tract[26]. The first bacteria to recover logarithmic growth were the aerobic natural constituents of the female genital tract flora.

Science demands that in order to attain perceived validation, a hypothesis must account for the exceptions. That douching causes BV does not account for the ability of 7 to 10 days of vaginal douching with povidone–iodine to achieve clinical and less often microbiologic cures in women diagnosed as having 'non-specific vaginitis/*Gardnerella vaginalis*/BV' (Federal Drug Administration (FDA) study files for Betadine Gel).

A second exception is identified in the Ness and co-workers' papers[24] which found that women who douched before or after sex did not have an increased risk of BV. This group reported a statistically higher frequency of sexual intercourse, on average, than other women in the study.

There is no argument as to the fact that women who douche, more likely than not, have an abnormal vaginal flora. What is in contention is what role douching has in the causation of such a condition and if so, how. What is done in the name of women's healthcare must have a valid foundation in more than epidemiologic studies. Executions should not be done on the basis of circumstantial evidence.

### REFERENCES

1. Fonck K, Kaul R, Feli F, *et al*. Sexually transmitted infections and vaginal douching in a population of female sex workers in Nairobi, Kenya. *Sex Transm Dis* 2001;77:271–5
2. Rajamanoharan S, Low N, Jones SB, Pozniak AL. Vaginal douching, bacterial vaginosis, ethnicity and use of genital cleaning agents: a case control study. *Sex Transm Dis* 1999;25:404–9
3. Holman C, Leventhal JM, Jones NM, Wang J, BV Study Group. Factors linked to bacterial vaginosis in nonpregnant women. *Am J Public Health* 2001;91:1664–70
4. Fiscella K, Franks P, Kendrick JS, Bruce PC. The risk of low birth weight associated with vaginal douching. *Obstet Gynecol* 1998;92:913–17
5. Gresenguet G, Kriess JK, Chapko MK, *et al*. HIV infection and vaginal douching in central Africa. *AIDS* 1997;11:101–6
6. Zhang J, Thomas G, Leybovich E. Vaginal douching and adverse health effects: a meta-analysis. *Am J Public Health* 1997;87:1207–11
7. LaRuche G, Messou N, Ali-Napo L, *et al*. Vaginal douching: association with lower genital tract infections in African pregnant women. *Sex Transm Dis* 1999;26:191–6
8. Ness RB, Soper DE, Holley RL, *et al*. Douching and endometritis: results from the PID evaluation and clinical health (PEACH) study. *Sex Transm Dis* 2001;28:240–5
9. Rosenberg MJ, Phillips RS. Does douching promote ascending infection. *J Reprod Med* 1992; 37:930–8
10. Stergachis A, Scholes D, Heidrich FE, *et al*. Selective screening for *Chlamydia trachomatis* infection in a primary care population of women. *Am J Epidemiol* 1993;138:145–53

11. Scholes D, Stergachis A, Ichikawa LE, *et al.* Vaginal douching as a risk factor for cervical *Chalmydia trachomatis* infection. *Obstet Gynecol* 1998;91:993–7
12. Scholes D, Darling JR, Stergachis A, *et al.* Vaginal douching as a risk factor for acute pelvic inflammatory disease. *Obstet Gynecol* 1993;81:601–6
13. Chow W-H, Darling JR, Weiss NS, *et al.* Vaginal douching as a potential risk factor for ectopic pregnancy. *Am J Obstet Gynecol* 1985;152:727–9
14. Darling JR, Weiss NS, Schwartz SM, *et al.* Vaginal douching and the risk of tubal pregnancy. *Epidemiology* 1991;2:40–8
15. Mueller BA, Luz-Jiminez M, Darling JR. Risk factors for tubal infertility: influence of history of prior pelvic inflammatory disease. *Sex Transm Dis* 1992;19:28–30
16. Wolner-Hansen P, Eschenbach DA, Paavonan J, Darling R, *et al.* Association between douching and acute pelvic inflammatory disease. *J Am Med Assoc* 1990; 263:1936–41
17. Monif GRG. The great douching debate: to douche or not to douche. *Obstet Gynecol* 1999; 94:630–1
18. Huck SW, Cormier WH, Bounds WG Jr. *Reading Statistics and Research*. New York: Harper & Row, 1974:Chapters 11,12,14
19. Larssen PG, Platz-Christensen JJ, Sunderstrom E. Is bacterial vaginosis a sexually transmitted disease? *Int J STD AIDS* 1991;2:362–4
20. Tchoudomirva K, Stanilova M, Garov V. Clinical manifestations and diagnosis of bacterial vaginosis in a clinic of sexually transmitted diseases. *Folia Med Plovdiv* 1998;40:34–40
21. Nilsson U, Hellberg D, Shoubnikova M, *et al.* Sexual behavior risk factors associated with bacterial vaginosis and *Chlamydia trachomatis* infection. *Sex Transm Dis* 1997;24:241–6
22. Joesoef MR, Wiknjosastro G, Norojono W, *et al.* Co-infection with chlamydia and gonorrhea among pregnant women with bacterial vaginosis. *Int J STD AIDS* 1996;7:61–4
23. Berger BJ, Kolton S, Zenilman JM. Bacterial vaginosis in lesbians: a sexually transmitted disease. *Clin Infect Dis* 1995;21:1402–5
24. Ness RB, Hillier SL, Richter HE, *et al.* Douching in relation to bacterial vaginosis, lactobacilli and facultative bacteria in the vagina. *Obstet Gynecol* 2002;100:765–72
25. Onderdonk AB, Delaney ML, Hinkson PL, Dubois AM. Quantitative and qualitative effects of douche reparations on vaginal microflora. *Obstet Gynecol* 1992;80:333–8
26. Monif GR, Thompson JL, Stephens HD, Baer H. Quantitative and qualitative effects of povidone-iodine liquid and gel on the aerobic and anaerobic flora of the female genital tract. *Am J Obstet Gynecol* 1980;137:432–8

RECEIVED 06/03/03; ACCEPTED 06/13/03
